# ASO Author Reflections: Cancer-Specific Mortality of Non-Muscle-Invasive Bladder Cancer

**DOI:** 10.1245/s10434-023-14301-w

**Published:** 2023-09-14

**Authors:** Aleksander Ślusarczyk

**Affiliations:** Department of General, Oncological and Functional Urology, Medical University of Warsaw, Warsaw, Poland

## Past

The majority of prognostic models and nomograms for non-muscle-invasive bladder cancer (NMIBC) classify individuals based on their risk of recurrence and progression.^1–4^ Exploration of cancer-specific survival (CSS) among NMIBC patients remains limited, owing to the utilization of surrogate endpoints and relatively infrequent occurrence of mortality attributed to bladder cancer (BC) within this cohort. Accurate insights into CSS prognostic determinants for NMIBC necessitate large cohorts within multicenter studies. Presently, comprehensive real-world data concerning survival outcomes and prognostic factors in NMIBC are deficient. We aimed to evaluate real-world CSS in NMIBC and to develop a prognostic nomogram based on identified risk factors.^5^

## Present

Our population-based analysis using the Surveillance, Epidemiology, and End Results database encompassed 98,238 patients with NMIBC who underwent transurethral resection of bladder tumor (TURBT) between 2004 and 2015. Our findings reveal significant disparities in cancer-specific mortality (CSM) across different NMIBC stages. Notably, T1HG and carcinoma in situ (CIS) were associated with the highest CSM rates within the observed follow-up of 124 months [interquartile range (IQR) 81–157], reaching 19.52% and 15.56%, respectively. The study also highlighted the relatively high risk of CSM for rarely diagnosed T1LG and TaHG tumors; the risk being approximately 10%. Our nomogram underlines the crucial relevance of T stage, grade and age in CSM prediction. Sociodemographic factors also influence the CSM, which underscores the disparity in oncological care and should alarm the healthcare providers and policymakers. Estimation of long-term CSM based on identified risk factors might help in patient counseling and aid clinical decision making regarding treatment intensification in ones and de-intensification in others.^5^

## Future

The anticipation of CSS, representing the paramount ultimate endpoint within oncological investigations, holds profound significance within the context of NMIBC. Future research endeavors pertaining to the prediction of progression-free and cancer-specific survival in NMIBC should be directed towards identifying strategies for precisely categorizing the subset of patients with the highest risk, necessitating intensified therapeutic interventions and meticulous surveillance. The development and implementation of innovative therapeutic paradigms remains an unmet need, particularly for individuals with high- and very high-risk NMIBC.^4^ Potentially synergistic adjuvant regimens after TURBT, accompanied by vigilant cystoscopic monitoring, might offer more effective bladder-sparing approaches, minimizing the need for radical cystectomy. The forthcoming outcomes from ongoing trials involving combinations of Bacillus Calmette–Guérin (BCG) vaccine and promising immunotherapies (e.g., immune checkpoint inhibitors) have the potential to reshape the current therapeutic landscape for high- and very high-risk NMIBC subgroups, thereby amplifying the significance of robust risk stratification strategies.^4^
